# Optical Fiber Performance for High Solar Flux Measurements in Concentrating Solar Power Applications

**DOI:** 10.3390/s25164973

**Published:** 2025-08-11

**Authors:** Manuel Jerez, Alejandro Carballar, Ricardo Conceição, Jose González-Aguilar

**Affiliations:** 1 Electronic Engineering Department, Universidad de Sevilla (E.T.S. de Ingeniería), C/Camino de los Descubrimientos s/n, 41092 Sevilla, Spain; mjerez@us.es; 2 High Temperature Processes Unit, IMDEA Energy, Av. Ramón de La Sagra, 3, Móstoles, 28935 Madrid, Spain; ricardo.conceicao@imdea.org (R.C.); jose.gonzalez@imdea.org (J.G.-A.)

**Keywords:** concentrating solar power, high-temperature optical fiber, radiometer, damage analysis, scanning electron microscopy, high-flux solar simulator

## Abstract

Extreme operating conditions in solar receivers of concentrated solar thermal power plants, such as high temperatures, intense irradiance, and thermal cycling, pose significant challenges for conventional sensors. Optical fibers offer a promising alternative for flux measurement in such environments, but their long-term performance and degradation mechanisms require detailed investigation and characterization. This work presents a proof of concept for high solar flux measurement by using optical fibers as photon-capturing elements and showcases the behavior and damage that these optical fibers undergo when exposed to relevant conditions, including temperatures over 600 °C and flux levels exceeding 400 kW/m^2^. Three fiber configurations, including polyimide and gold-coated fibers, were tested at a high-flux solar simulator and analyzed via scanning electron microscopy to assess structural integrity and material degradation. Results reveal significant coating deterioration, fiber retraction, and thermal-induced stress effects, which impact measurement reliability. These findings provide essential insights for improving the durability and accuracy of optical fiber-based sensing technologies in concentrating solar energy.

## 1. Introduction

Optical fiber sensors represent a critical technological advancement in monitoring and sensing capabilities in extreme environmental conditions, offering unprecedented reliability and performance where traditional sensing technologies struggle [1,2]. The unique characteristics of optical fiber-based sensing systems, such as immunity to electromagnetic interference, lightweight design, and ability to withstand harsh operational environments [3], have positioned them as transformative technologies across multiple scientific and industrial domains. From deep-sea exploration [4], seismic movements [5], or volcanic monitoring [6] to aerospace [7,8] and high-temperature industrial processes [9,10,11,12], optical fiber sensors provide real-time, high-precision measurements in environments characterized by extreme temperatures, pressures, chemical exposures, and radiation levels [1,2,13].

In parallel to the progress of optical fiber sensing technologies, the global energy landscape has undergone a transformative shift towards renewable energy sources [14,15,16,17], with particular emphasis on sustainable and efficient power generation technologies. This paradigm shift has brought concentrated solar thermal (CST) power plants to the forefront, presenting unique challenges in operational monitoring and performance optimization [18,19,20]. The development of sensors enables predictive maintenance strategies, reduces downtime, and optimizes energy production by providing real-time, high-resolution data about the complex thermal and mechanical dynamics [21] within these advanced energy generation systems.

The intricate nature of CST systems demands sophisticated sensing technologies capable of providing real-time, precise measurements across a range of extreme environmental conditions, including radiation levels around 750 kW/m^2^ (with peaks over 1.5 MW/m^2^) and temperatures rising up to 1000 °C with gradients that can exceed 500 °C [22,23,24]. The quantification of concentrated flux in diverse CST configurations is essential for plant condition monitoring; knowledge of both the incident radiation at the receiver and its spatial distribution enables production optimization and heliostat tracking anomaly detection. Additionally, receiver temperature monitoring ensures the control of critical operational parameters, maintaining material integrity while preserving energy production process efficiency [25,26]. Despite the importance of this task, the measurement of concentrated solar flux in CSP systems presents significant challenges due to the extreme conditions of temperature and radiation that sensors must bear. Traditional methodologies based on Gardon gauges present two substantial limitations: first, the occupation of non-negligible surface area in the receiver, shadowing or reducing the effective area used for energy generation, and second, the necessity for cooling systems that introduce additional system complexity and more maintenance requirements [27,28]. Consequently, the implementation of alternative sensing technologies becomes imperative for accurate concentrated flux measurement while maintaining system integrity. Various alternative techniques to the traditional ones have been presented for measuring flux in CSP receivers [29,30], including the use of radiometers in novel or mobile setups [31], cameras [32,33,34], or, more recently, the use of artificial intelligence [35,36].

The use of optical fibers as radiation collectors in solar receivers in CSP applications emerges as a potential option to enhance plant monitorization. Nevertheless, the path to widespread deployment necessitates rigorous proof of concept and comprehensive characterization of potential damage mechanisms [37,38,39,40]. The operational conditions of CSP environments, characterized by high temperatures, thermal cycling, mechanical stress, and prolonged exposure to concentrated solar radiation, demand meticulous validation of sensor performance and long-term reliability. Key parameters under scrutiny include changes in sensitivity, accuracy, as well as structural integrity damage and potential degradation of fiber materials.

To conduct an initial characterization of a high solar flux measurement system using optical fibers as light sensors, a proof-of-concept experiment was performed in a high-flux solar simulator (HFSS) at IMDEA Energy, Móstoles, Spain, whose results will be briefly presented. After the testing campaign, a detailed examination of radiation-exposed fibers was conducted utilizing scanning electron microscopy (SEM) to perform a comprehensive analysis of degradation mechanisms, which will be addressed in this work.

The remainder of this paper is organized into four sections: Section 2 introduces the use of optical fibers as light-capturing elements by performing a proof of concept for high solar flux measurements, as well as the experimental setup utilized for the exposition towards radiation; Section 3 presents the experimental results for the obtained measurements and the optical fibers damage assessment, as explored utilizing SEM. Finally, Section 4 outlines the main conclusions.

## 2. Proof of Concept for High Solar Flux Measurements

**Figure 1:**
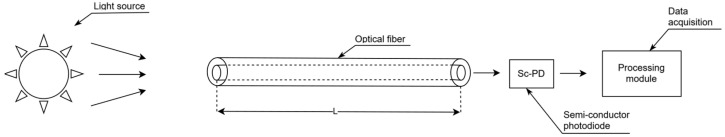
Block diagram of the conceptual optical fiber and photodiode-based radiometer.

Previous research demonstrates the implementation of an optical fiber, in conjunction with a photodiode and calibration algorithm, functioning as a radiometer for monitoring Direct Normal Irradiance (DNI) with accuracy comparable to commercial devices [41]. The use of optical fibers as the collector element for solar radiation has a wide range of benefits. The first advantage is that it is a passive element requiring no power supply, simplifying its assembly and operation. Additionally, it possesses electromagnetic noise immunity, which is crucial in high-radiation environments; it has a low cost and does not suffer from physical issues, such as corrosion or oxidation that affect traditional electronic systems. The combination of this device with a semiconductor photodiode enables near-zero response time and extremely high sensitivity, along with the capability to decouple the measurement location from the radiation collection point, isolating it from extreme conditions. Figure 1 shows the conceptual block diagram for the optical fiber-based radiometer.

In this context, the implementation of a new measurement system is proposed based on optical fiber and photodiode technology for high solar flux measurement. The system operates by positioning the optical fiber oriented towards solar radiation, as depicted in Figure 1, capturing and transmitting light to a semiconductor photodiode where the captured optical power is quantified and subsequently converted to flux measurements through a calibration algorithm [41]. The system presents multiple significant advantages. Firstly, the spatial efficiency, as the exposed fiber tip occupies a surface area several magnitudes orders smaller than traditional Gardon gauges, which reduces the interference with the receiver operation and the integration of the sensor within the receiver structure. The second main advantage is the spatial decoupling, allowing for the isolation of the measurement electronics from the extreme conditions suffered in the CSP tower plant.

Building upon these findings, the objective is to adapt the radiometric system in [41] for high solar flux measurement. Despite preliminary success, significant technological challenges remain to be addressed, specifically the thermal requirements, having to deal with sustained temperature resistance of around 800 °C with peaks up to 1000 °C in real CSP generation plants, while measuring concentrated flux up to 1.5 MW/m^2^. These extreme operational conditions require the use of special optical fiber designs.

### 2.1. “Ad-Hoc” Optical Fiber Cables for Radiometric Measurements in Harsh Environments

Typically, most commercial optical fibers are mostly composed of silicon oxide, which exhibits thermal stability up to approximately 1200 °C. Above this temperature, the material begins to crystallize, resulting in a significant performance degradation. Additionally, traditional optical fibers (as those designed for telecommunications) are encapsulated in polymeric materials that substantially limit their operational temperature to approximately 100 °C [2,37,40]. For applications with stringent temperature requirements such as those in concentrated solar energy, recent advancements have introduced sapphire-based optical fibers, which operational capabilities exceed 2000 °C [42,43,44,45], but at high cost.

**Figure 2:**
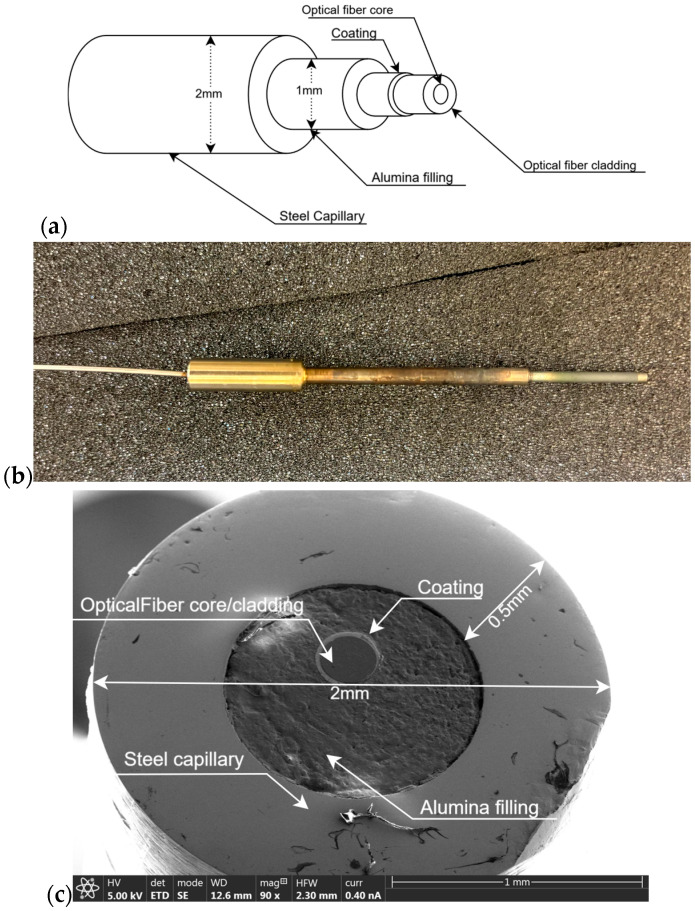
(**a**) Scheme of the optical fiber cable end used for high solar flux measurement; (**b**) picture of the assembly of the optical fiber cable; (**c**) scanning electron micrograph of the tip of the “ad hoc” optical fiber cable for CSP applications.

In order to perform high solar flux measurements in CSP applications, an “ad hoc” optical fiber cable was designed and developed to withstand the extreme environment. Figure 2 illustrates the optical fiber structure developed for this purpose and a scanning electron micrograph of its tip, which receives the high-intensity sunlight. A high-temperature-resistant coating encapsulates the optical fiber, providing protection against extreme environments. The high-temperature optical fiber is positioned within a steel capillary, with an insulating material, alumina, strategically placed between the fiber and the capillary as a filling medium. This configuration keeps the structural integrity of the entire device. However, this configuration is specifically implemented only for the final 2 cm exposed to radiation. The overall assembly has a total thickness of 2 mm, with the fiber diameter varying according to the specific design configurations implemented.

During the experimental campaign, three optical fiber cables have been utilized. The three optical fiber cables have the same structure presented in Figure 2, but they contain different high-temperature optical fibers inside. Specifically, the first one features a core diameter of 50 μm and an outer diameter of 125 μm, with a polyimide coating (denoted as 50/125PI). The second one shares the same coating type but exhibited larger dimensions, with a core diameter of 200 μm and an outer diameter of 220 μm (denoted as 200/220PI). The last fiber has identical dimensional characteristics to the second one, but it is distinguished by a gold coating (thus denoted as 200/220Au). Regarding core and cladding composition, core of the fiber was always composed of pure silica; while cladding was germanium doped silica in polyimide coated fibers and fluorine doped silica in the gold coated specimen. All optical fiber cables were manufactured by the Engionic group, Berlin, Germany (https://www.engionic.de/, accessed on 10 May 2025). A summary of the three high-temperature optical fibers used to build the “ad hoc” optical fiber cables to perform the high solar flux measurements can be found in Table 1.

According to the manufacturer, the developed optical fibers demonstrate stable operational performance up to 700 °C, with the capacity to withstand temperature peaks reaching 1000 °C. Although these characteristics marginally meet CSP real operating condition requirements, they are more than adequate for conducting the proof-of-concept experiment in the HFSS, which does not exceed temperatures of 600 °C.

### 2.2. Experimental Setup

For the proof of concept of high solar flux measurements, an HFSS was used as the light source. The high-flux solar simulator installed at IMDEA Energy, designated as KIRAN-42 [46], comprises two distinct rooms: one for the test bench and another housing the simulator itself. The HFSS incorporates seven short-arc xenon lamps arranged in a hexagonal configuration around a central lamp. Each lamp is located in one of the focal points of an ellipsoidal reflector and conveniently oriented to have a joint single second focal point. Each lamp operates at an electrical power of 6 kW, yielding a total electrical power capacity of 42 kW, which translates to an aggregate radiative power output of 14 kW at the testing bench plane. The system can achieve a peak flux density of 3.6 MW/m^2^ with a sustained flux density of 2.7 MW/m^2^. Individual cooling systems are integrated into each lamp assembly to ensure optimal heat dissipation and lamp lifetime.

**Figure 3:**
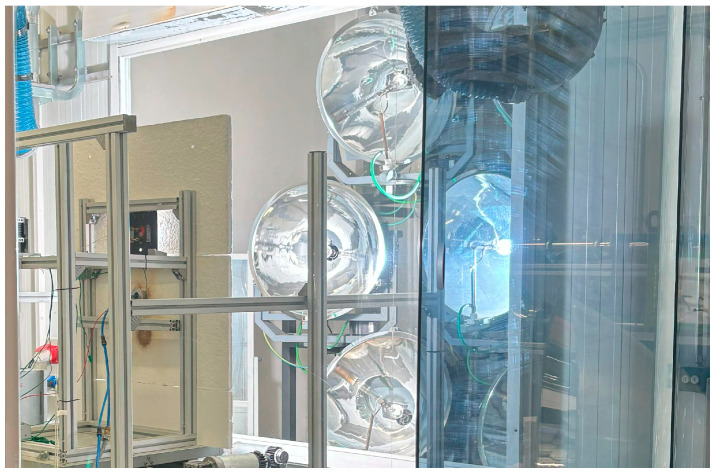
The 42 kW high-flux solar simulator at the IMDEA Energy facility viewed through the safety observation window, showing the testing bench and the lamp array assembly with one xenon arc lamp being used.

The adjacent chamber for the test bench, separated by a retractable blind (withdrawn during experimental procedures), houses the positioning table equipped with three motors enabling precise XYZ-axial mobility with 1 mm resolution. This testing platform accommodates both the designed system under test and a permanently mounted Gardon radiometer for flux measurements. System control operations are conducted externally to both chambers, enabling management of lamp activation/deactivation, positioning table movement, and cooling/gas extraction systems. Figure 3 depicts an external view of the HFSS, featuring the control equipment array in the foreground. Behind the observation window, the testing bench and the seven-lamp array comprising the simulator are visible, one of them being turned on.

**Figure 4:**
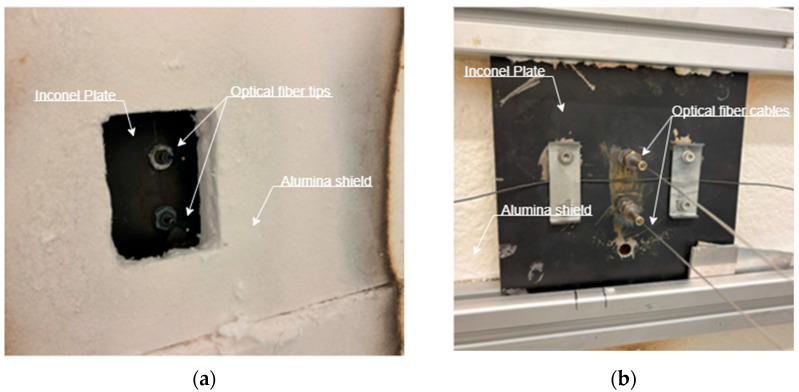
Optical fiber assembly for the high solar flux measurement: (**a**) radiation incident surface of the Inconel plate; and (**b**) posterior view of the configuration.

The experimental configuration for the high solar flux measurement using optical fibers is shown in Figure 4. It employed an Inconel plate fitted with two MK-125-A waterproof connectors, through which the optical fiber cable was threaded for radiation exposure. The plate was mounted on a structural framework designed to maintain perpendicularity relative to the incoming radiation, ensuring precise alignment of the optical fiber. The Inconel plate, susceptible to thermal deformation at the operational temperatures encountered, was shielded by an alumina plate, featuring a small aperture or “window” as the sole exposed region to incident radiation.

## 3. Experimental Results

### 3.1. High Solar Flux Measurements

The experimental protocol to perform high solar flux measurements consisted of the following sequence: after ignition and stabilization of the central lamp and subsequent flux measurement using a Gardon radiometer, each fiber was positioned at the focal point for approximately 7 min to study the temporal evolution of measurements. Following the initial exposure, upon removing the fiber from the focal point, a second lamp was activated. Once stable conditions were achieved, the fiber was repositioned at the focal point for radiation measurement. However, this secondary exposure phase was necessarily limited to a few seconds due to the observable red heat coloration and consequent deformation of the Inconel structure. During the experiments, the optical fibers were subject to extreme temperature conditions, with temperatures around 400 °C during single-lamp experiments, increasing up to 600 °C when two lamps were activated.

**Figure 5:**
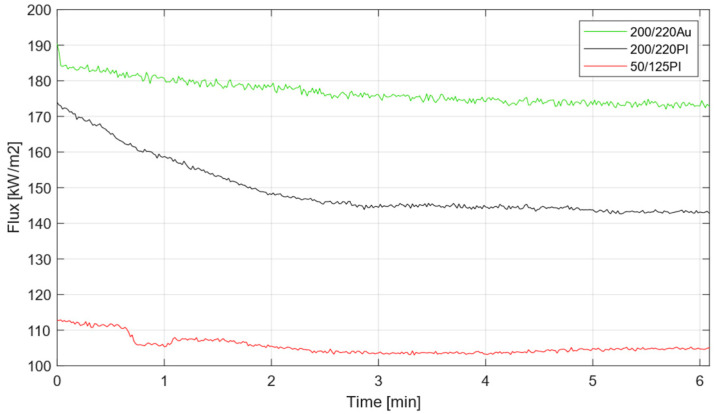
High solar flux measurements with 200/220Au (green), 200/220PI (black), and 50/125PI (red) optical fiber cables.

The high solar fluxes measurements obtained by the optical fiber and photodiode-based radiometer are shown in Figure 5, where the measurements acquired and calibrated following the algorithm described in [41] with the three optical fiber cables are compared. In this experiment, the objective was to study the evolution of the flux and reach the stabilization point, which would be reached around 5 min after the positioning of the fiber in the focal point. The peak fluxes obtained by the fibers were below 200 kW/m^2^ and decayed along the time, in contrast with the flux measurement provided by the Gardon radiometer. Theoretically, these measured flux values should be equal, as the radiative conditions in both samples were the same, but this was not the case, as the values registered by the gauge were between 300 kW/m^2^ and 310 kW/m^2^. This measurement discrepancy was further amplified upon activation of a second lamp, where the Gardon radiometer readings exceeded 400 kW/m^2^, while the fiber measurements remained below 220 kW/m^2^. Among the different optical fibers, the gold-coated one demonstrated better behavior, providing higher peak and permanent measurements. The experimental results obtained demonstrate the viability of using the proposed radiometer configuration based on the optical fiber as solar radiation collector, although some challenges remain to be addressed.

**Figure 6:**
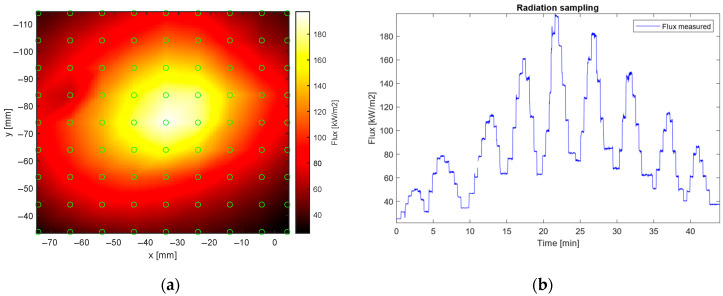
(**a**) Flux measured using optical fiber-based radiometer configuration, moving the fiber around the focal point, and (**b**) power levels measured during the experiment.

Next, using the 200/220Au optical fiber cable for a matrix of punctual measurements, a surface spatial distribution for the emitted radiation from the HFSS was performed. Results are presented in Figure 6. To achieve that, the optical fiber tip was placed successively in different positions around the focal point, marked with green dots in Figure 6a, resulting in the different measurements shown in Figure 6b. Then, an interpolation was carried out with the purpose of obtaining the surface flux distribution provided by the HFSS. This spatial mapping of surface flux is crucial for concentrated solar power (CSP) receivers, as it allows for a detailed understanding of the energy input over the receiver surface. This information is essential for optimizing receiver design, ensuring efficiency and reducing gradients, prolonging the lifespan of the receiver [25,26].

While the observed behavior under dual lamp operation was anticipated, given that the secondary lamp’s light emanates at an angle exceeding the optical fiber’s field of view (as determined by its numerical aperture), the fiber’s performance under single lamp operation deviated significantly from both ideal and expected responses. Although substantial flux values were recorded, these measures remained lower than those obtained from the Gardon radiometer. Multiple factors may have contributed to this discrepancy, including fiber attenuation for non-perpendicular angles of incidence; suboptimal positioning and focal alignment of the fiber; age-related degradation and physical damage to the fiber; and temperature-dependent measurement effects.

Regarding the latter hypothesis concerning temperature influence on measurements, it is imperative to address the observed dynamics during flux measurements across various experimental trials. A consistent phenomenon was documented across all experiments: a notable decrease in measured values was observed from the initial moment of fiber placement at the focal point until measurement stabilization was achieved, as shown in Figure 5 in the flux measurement with the 200/220PI optical fiber cable (black line). The main explanation for this fact is the relationship of the measurement with the temperature until it reaches thermal stability. The lamp is focused on the Gardon until it reaches its maximum value of light intensity and temperature, so that once the fiber is moved to the focus, a heating period begins (in general, of the Inconel plate and the connection with the fiber), which coincides with the time in which the measurement of the fiber is falling.

A technical remark should be made concerning the fiber’s core and cladding materials. As noted before, the polyimide specimens were germanium-doped silica, whereas the gold-coated fiber featured fluorine-doped silica in its cladding. According to the literature [47] on dopant effects in optical fibers, fluorine-doped fibers deliver the highest radiation tolerance of any common dopant: they suppress refractive index perturbations and enhance thermal annealing recovery, which could have contributed to the difference between gold and polyimide coated fibers. This evidence strongly favors the use of gold-coated fibers—not only for the resistance of their coating but also for the optical fiber composition.

The observed phenomena led us to the necessity of investigating if this temperature effect also had a visible impact on the fiber itself.

### 3.2. Damage Analysis of Optical Fibers Exposed to Concentrated Solar Radiation

The following results present the microscopic analysis of the optical fiber cable tips exposed to the concentrated solar radiation, utilizing a Thermofisher Teneo high-resolution SEM located at CITIUS, Seville, Spain (https://citius.us.es/, accessed on 10 May 2025). Comparative analyses were conducted for the optical fibers cables, before and after radiation exposition, with the purpose of evaluating radiation-induced effects. In addition to the optical fibers tested at the HFSS, two more experiments were executed at CITIUS, comparing the condition of new optical fibers before and after high-temperature exposure in a tubular furnace. These additional results are described in Appendix A.

**Figure 7:**
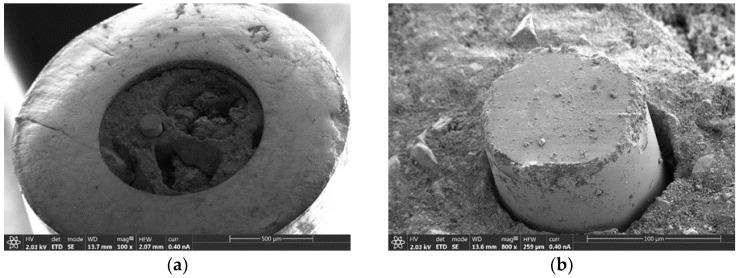
Tip of 50/125PI optical fiber cable: (**a**) general structure of the capillary and (**b**) detail of the tip of the optical fiber.

The initial probe analyzed by the SEM was the 50/125PI. This particular fiber exhibited complete signal loss after the testing campaign in the HFSS, registering null optical power measurements despite direct exposure to the lamp array after the second lamp was activated. While SEM analysis was conducted to determine possible causes of functionality loss, the micrographs did not provide any conclusion regarding the failure. Figure 7a presents a general view of the optical fiber cable termination, clearly delineating the external metallic capillary, insulating material infill (alumina-like), and the protruding optical fiber tip. Macroscopic structural damage appears limited to crater formations in the infill material, which theoretically should not impair the fiber’s optical power capture and transmission capabilities.

Figure 7b provides a detailed examination of the 50/125PI fiber termination, revealing two significant observations. Primary examination of the fiber itself indicates wear patterns and peripheral fracturing of the fiber cladding, accompanied by surface contamination deposits. Additionally, a void space is observed between the fiber and alumina interface, corresponding to the expected location of the polyimide coating, suggesting thermal volatilization of this component. This latter phenomenon will be consistently observed in subsequent analyses.

**Figure 8:**
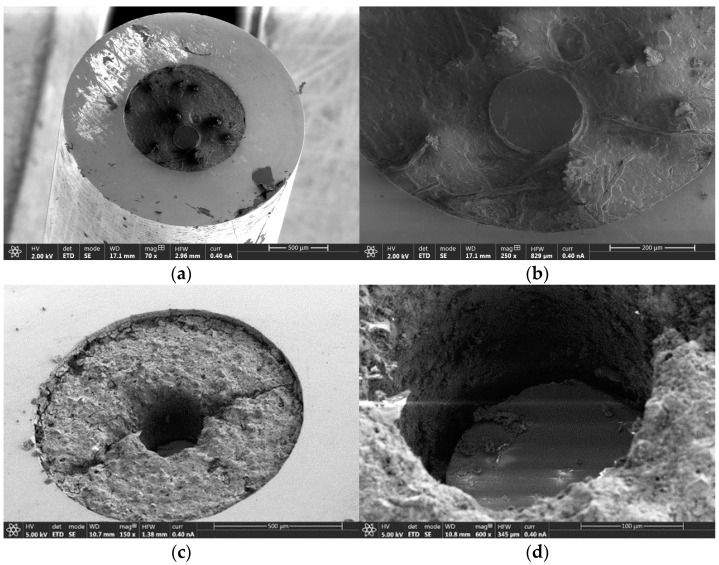
Tip of the 200/220PI optical fiber cable: (**a**) general structure of the capillary before radiation exposure; (**b**) detail of the tip of the optical fiber before radiation exposure; (**c**) general structure of the capillary after radiation exposure; (**d**) detail of the tip of the optical fiber after radiation exposure.

The next analyzed sample corresponds to the 200 μm core diameter optical fiber with a polyimide coating, that is, 200/220PI optical fiber cable. Figure 8 shows the SEM analysis to the 200/220PI optical fiber cable before and after radiation exposition. Figure 8a,b present the SEM images for the 200/220PI optical fiber cable before radiation exposition. The observation of these images demonstrates that the optical fiber maintains coplanar alignment with the surrounding structure, with no visible cracking in the alumina component and no apparent gap between the optical fiber and the infill material. This latter observation confirms the intact presence of the polyimide coating in the optical fiber before radiation exposition. However, after radiation exposure, the most significant observation from Figure 8c,d is that the optical fiber has retracted from the surrounding structural surface, in contrast to the previous example where it had risen. As before, a void space is evident between the fiber core and the metallic capillary infill, indicating the loss or degradation of the polyimide coating. Another distinction from the previous fiber is that the infill material exhibits relatively less structural damage, presenting only minor transverse cracking patterns.

**Figure 9:**
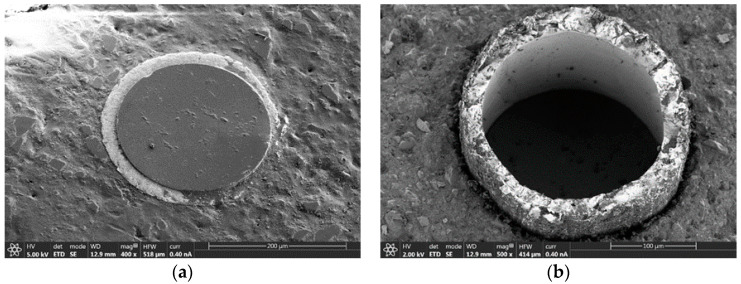
Tip of two 200 μm core diameter and gold sheath fiber: (**a**) 200/220Au fiber never used; (**b**) 200/220Au fiber after experiments in solar simulator.

The final analysis specimen from the HFSS experiments consists of the 200/220Au optical fiber cable, the one with gold coating, whose results are presented in Figure 9. The primary distinguishing characteristic from previously analyzed specimens is the structural integrity of the gold coating, as illustrated in Figure 9b, which maintains its cohesion in contrast to the degradation observed in polyimide-coated specimens. Furthermore, the gold coating exhibits greater thickness compared to the void spaces observed in previous samples where coating degradation occurred.

Figure 9a shows the reference specimen 200/220Au before radiation exposure. Comparative analysis between Figure 9a,b (reference specimen after radiation exposure) reveals distinct morphological differences. In this instance, the alumina infill material exhibits various crack formations, indicating thermal-induced stress patterns. However, the preservation of the gold coating’s structural integrity demonstrates its superior thermal stability compared to polymer-based coating systems under high-flux radiation conditions. Additionally, Figure 9b discovers a deep fiber’s retraction from the surrounding structural surface.

**Figure 10:**
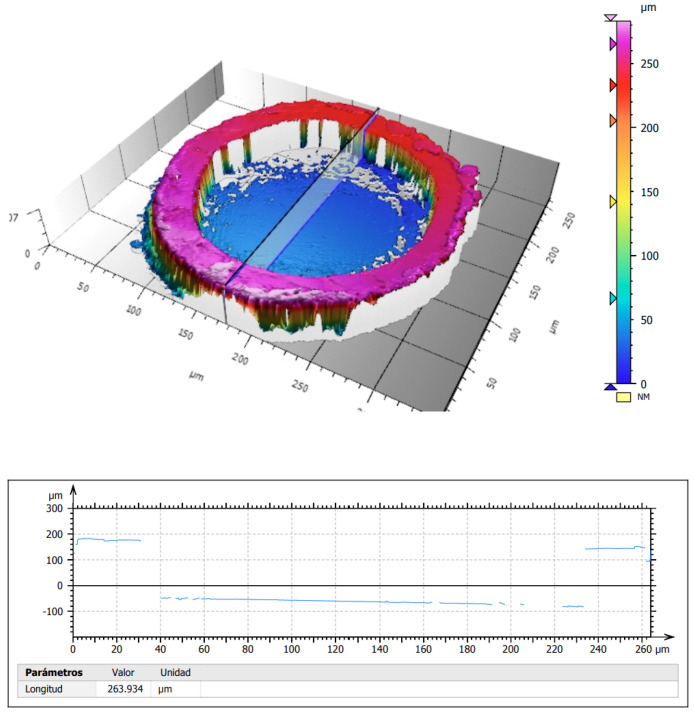
Surface analysis of the 200/220Au optical fiber cable after usage at the solar simulator.

To conduct a more detailed analysis of the fiber’s retraction, the 200/220Au optical fiber cable termination was examined using a Sensofar S Neox (Terrassa, Catalonia, Spain) onfocal interferometric microscope. As detailed in Figure 10, the displacement of the fiber tip relative to the gold coating exceeds 240 μm, resulting in an inter-layer distance greater than the fiber tip’s own diameter of 220 μm. This significant retraction phenomenon can potentially result in a reduction in the field of view due to an “overshadowing effect” on the fiber core, as the walls formed around the fiber prevent light coming from wider angles to be coupled. The magnitude of this displacement suggests significant thermal–mechanical stress effects during exposure, with potential implications for optical collection efficiency and overall sensor performance. This geometric modification represents a critical consideration in the design of optical fiber-based sensing systems operating under high-flux radiation conditions.

The conducted analysis shows that extreme radiation and temperature conditions significantly alter the fiber’s geometry and structural integrity, directly impairing its ability to measure radiant flux accurately. An isolated study of the thermal effects on the fiber was conducted and is included in Appendix A.

### 3.3. Discussion

The microscopic examination of optical fiber cables exposed to high-intensity radiation and elevated temperatures revealed several degradation mechanisms, potentially impacting sensing performance. Across all tested configurations, structural and material alterations were consistently observed when compared to unused references.

The 50/125PI fiber, despite full exposure to the solar simulator, showed complete signal loss. However, SEM analysis failed to pinpoint a definitive cause. Observable features included peripheral cladding fractures and polyimide volatilization, evident from voids between the fiber and surrounding infill. Interestingly, no major macroscopic damage was found that would directly account for total transmission failure, suggesting internal or degradation beyond SEM observations. Similarly, 200/220PI fiber demonstrated coating disappearance, but with comparatively less structural damage in the alumina infill. Retraction of the fiber from the surrounding capillary and void formation confirm thermal decomposition of the polyimide layer, which was supported by the experiments described in Appendix A.

In contrast, the 200/220Au fiber, featuring a gold coating, retained structural integrity post-exposure. The coating’s thickness and thermal resilience proved advantageous, avoiding the degradation patterns noted in polymer-coated fibers. Nevertheless, alumina cracking and significant fiber retraction were detected. The geometric displacement observed in both 200/220PI and 200/220Au fibers introduces an “overshadowing effect”, which could narrow the effective field of view and reduce coupling efficiency, leading to potential power loss that could explain the discrepancy in the measurements compared to the Gardon radiometer.

Overall, results confirm that both radiation and thermal loads introduce critical deformations and detachment phenomena at the fiber tip, especially in polymer-coated configurations, which proved to not withstand temperatures around 600 °C. These effects, if unmitigated, compromise optical transmission and measure reliability. Gold-coated fibers offer superior thermal stability but are not immune to mechanical stress and misalignment.

## 4. Conclusions

In this work, a proof of concept for measuring concentrated solar flux was conducted to evaluate the feasibility of utilizing optical fibers as radiometers in CSP tower facilities. Three optical fiber cable types, comprising different materials and geometries, were subjected to radiation from a xenon arc lamp, experiencing flux values exceeding 400 kW/m^2^ and temperatures around 550 °C. Throughout the experiments, the viability of measuring concentrated solar flux was demonstrated, although significant challenges remain to be addressed. Notable among these challenges is the necessity to expand the fiber’s field of view, currently constrained to a few degrees by numerical aperture, to encompass radiation from the entire heliostat field. Furthermore, mechanisms must be developed to mitigate the temperature dependence of the measurements.

Following the testing campaign in HFSS, the fiber tips were analyzed by SEM, which revealed substantial structural modifications despite exposure to temperatures within their design specifications. All specimens exhibited alterations in the alumina used to fill the metallic capillary; however, this should not affect the measurements, as this material serves solely as a thermal insulator. Furthermore, regarding material considerations, multiple experiments demonstrated that polyimide is not a suitable material for optical fiber coating in our application. This conclusion is based not only on worse behavior during testing campaign, but also on the material’s apparent degradation when operating at elevated temperatures, even during brief exposure periods. In this context, it was established that, between the two currently available options, gold presents a superior alternative for optical fiber coating, maintaining its structural integrity under extreme conditions.

In addition to the capillary structure modifications, the optical fibers themselves underwent alterations, exhibiting either retraction or protrusion by a non-negligible distance relative to the system’s surface; in the latter case, this displacement may have contributed to the observed power losses through core shadowing effects.

Future work will be focused on investigating various alternatives to protect the fiber tip from extreme conditions that appear to cause degradation, while simultaneously reducing the temperature dependence of the measurements. These solutions encompass the implementation of lenses, filters, or other optical devices, as well as the potential installation of fibers in alternative support structures and materials to minimize heat induction from these components.

## Figures and Tables

**Table 1:** Design parameters of the high-temperature optical fibers used for building optical fiber cables.

Core Diameter [μm]	Cladding Diameter [μm]	Numerical Aperture	Coating Type	Tag Used as a Reference
50	125	0.22	Polyimide	50/125PI
200	220	0.22	Polyimide	200/220PI
200	220	0.22	Gold	200/220Au

**Figure A1:**
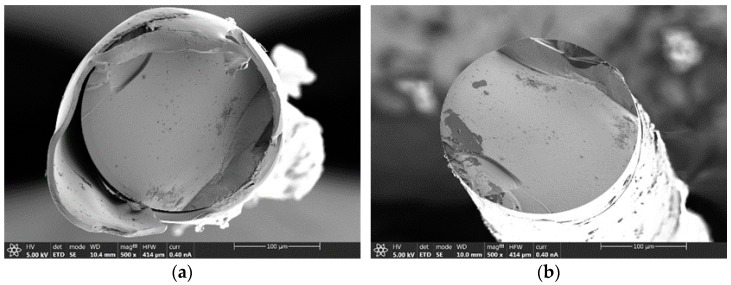
Tip of a 200/220PI optical fiber, i.e., 200 μm core diameter and polyimide sheath fiber without capillary: (**a**) before furnace treatment; and (**b**) after furnace treatment at 600 °C.

**Figure A2:**
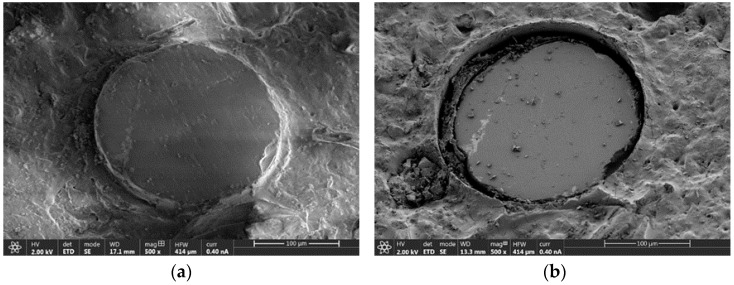
Tip of a 200/220PI optical fiber cable, i.e., a 200 μm core diameter, 220 μm cladding diameter, polyimide sheath fiber, alumina infill, and steal capillary: (**a**) before furnace treatment and (**b**) after furnace treatment at 600 °C.

## Data Availability

The raw data supporting the conclusions of this article will be made available by the authors on request.

## Abbreviations

The following abbreviations are used in this manuscript:

CSP	Concentrated Solar Power
HFSS	High-Flux Solar Simulator
IMDEA	Instituto Madrileño de Estudios Avanzados
SEM	Scanning Electron Microscopy
CITIUS	Centro de Investigación, Tecnología e Innovación de la Universidad de Sevilla

## Appendix A. Temperature-Induced Damage Assessment

Previous analyses demonstrate that extreme radiation and temperature conditions significantly impact fiber geometry and structural integrity, directly affecting flux measurement capabilities. However, as the fibers were simultaneously exposed to both high radiation and temperature conditions, the individual contributions of these factors to fiber performance degradation remained unclear.

To isolate temperature effects on fiber terminations, an experimental protocol was implemented wherein two pristine optical fibers were initially examined via SEM, subsequently subjected to thermal exposure at 600 °C for 15 min in a tubular furnace, and then re-examined microscopically. This investigation utilized two specimens: a 200/220PI optical fiber cable, i.e., a 200 μm core diameter fiber with polyimide coating featuring the complete mechanical assembly (previously shown in Figure 8), and an unprocessed sample of identical specifications (fiber and coating material) without the alumina and metallic capillary structure.

The polyimide degradation phenomenon is particularly pronounced in the unprocessed 200/220PI optical fiber specimen exposed to high temperature at 600 °C for 15 min, consisting solely of the optical fiber and polyimide coating. Figure A1a,b demonstrate complete dissolution of the coating material while the fiber core maintains structural integrity. These observations suggest that thermal exposure alone can initiate significant degradation of protective coatings, potentially contributing to the structural modifications observed in specimens subjected to combined thermal and radiation exposure in solar simulator testing.

For the mechanically processed optical fiber cable, notable morphological changes were observed despite the relatively brief thermal exposure period (15 min) at temperatures within the manufacturer’s specified operational range, as evidenced in Figure A2a,b. Primary observations include slight fiber recession relative to the supporting structure, transitioning from a position slightly above the alumina surface to below it. Additionally, the thermal exposure enhanced the delineation between the three primary components (fiber, polyimide coating, and alumina). Figure A2b reveals preferential degradation of the polyimide coating between the fiber and alumina, exhibiting significant fracturing patterns indicative of the complete degradation observed in IMDEA-tested specimens.
